# A Bilateral Diaphragmatic Paralysis Post-COVID-19 Infection: A Case Report and a Review of the Literature

**DOI:** 10.7759/cureus.35791

**Published:** 2023-03-05

**Authors:** Sallam Alrosan, Vincent M Lem, Mohammad Abu-Jeyyab

**Affiliations:** 1 School of Medicine, University of Kansas Medical Center, Kansas, USA; 2 Intensive Care Unit, Midwest Pulmonary Consultants, Kansas, USA; 3 School of Medicine, Mutah University, Amman, JOR

**Keywords:** dyspnoea, phrenic nerve, electromyography, diaphragmatic paralysis, : covid-19

## Abstract

The diaphragm is the essential respiratory muscle, and damage can significantly impede a human's capacity for blood oxygenation. During inspiration, the diaphragm domes permit the pleural cavity to expand. Whenever this process is disrupted, it results in decreased thoracic expansion and, as a result, hypoventilation. The phrenic nerve innervates the diaphragmatic muscle via the cervical nerve roots C3, C4, and C5. Diaphragmatic paralysis is a multifactorial consequence caused by trauma, neurogenic diseases, infections, inflammatory responses, and chest operative surgery, with the last being the most prevalent causative factor.

Here, we are describing the case of a 52-year-old male patient who has had ongoing dyspnea for months after contracting COVID-19 in December 2021, despite the remission of his previous COVID-19 pneumonia in 2020. An X-ray of the chest revealed no diaphragm elevation, whereas electromyography verified diaphragm impairment. On the conservative treatment plan, he reported persistent dyspnea following a period of pulmonary rehabilitation. To a lesser extent, it is advised to wait at least one year to see if there is any reinnervation, which could benefit his lung capacity.

COVID-19 has been linked to many systematic diseases. As a result, COVID-19 will not be restricted to its inflammatory effect on the lungs. In other words, it is a multi-organ systematic syndrome. One of these effects is diaphragm paralysis, which should be considered a post-COVID-19 disease. However, there is a need for more literature to support physicians as guidelines for neurological conditions related to COVID-19 infection.

## Introduction

Since its identification, the severe infection of the acute respiratory syndrome coronavirus [1] (SARS-CoV-2) has caused several pulmonary and non-pulmonary problems, including those of the cardiovascular and neurologic systems [1-3].

Lower respiratory tract involvement is a prominent hallmark of infection with the SARS-CoV-2 virus, most notably respiratory failure owing to viral pneumonitis [2]. However, long-term effects are appearing as the COVID-19 pandemic unfolds. Among them are neurological involvement [1,2] and chronic lung disease, particularly pulmonary fibrosis. This case report describes the bilateral diaphragmatic paralysis condition of a patient infected with SARS-CoV-2 and the likely contributing causes and takeaways from this unusual case [3,4].

## Case presentation

A 52-year-old male patient presented to the pulmonary clinic in 2020, a month after being diagnosed with COVID-19 disease for the second time - the first episode was at the end of 2019, which resolved without complications - by a nasal polymerase chain reaction swap with symptoms including myalgia that had woken him up in the night, a fever of 103 °F later, which reached 105 °F, and fatigue that was not controlled with ibuprofen or acetaminophen; in addition, he developed shortness of breath.

**Figure 1:**
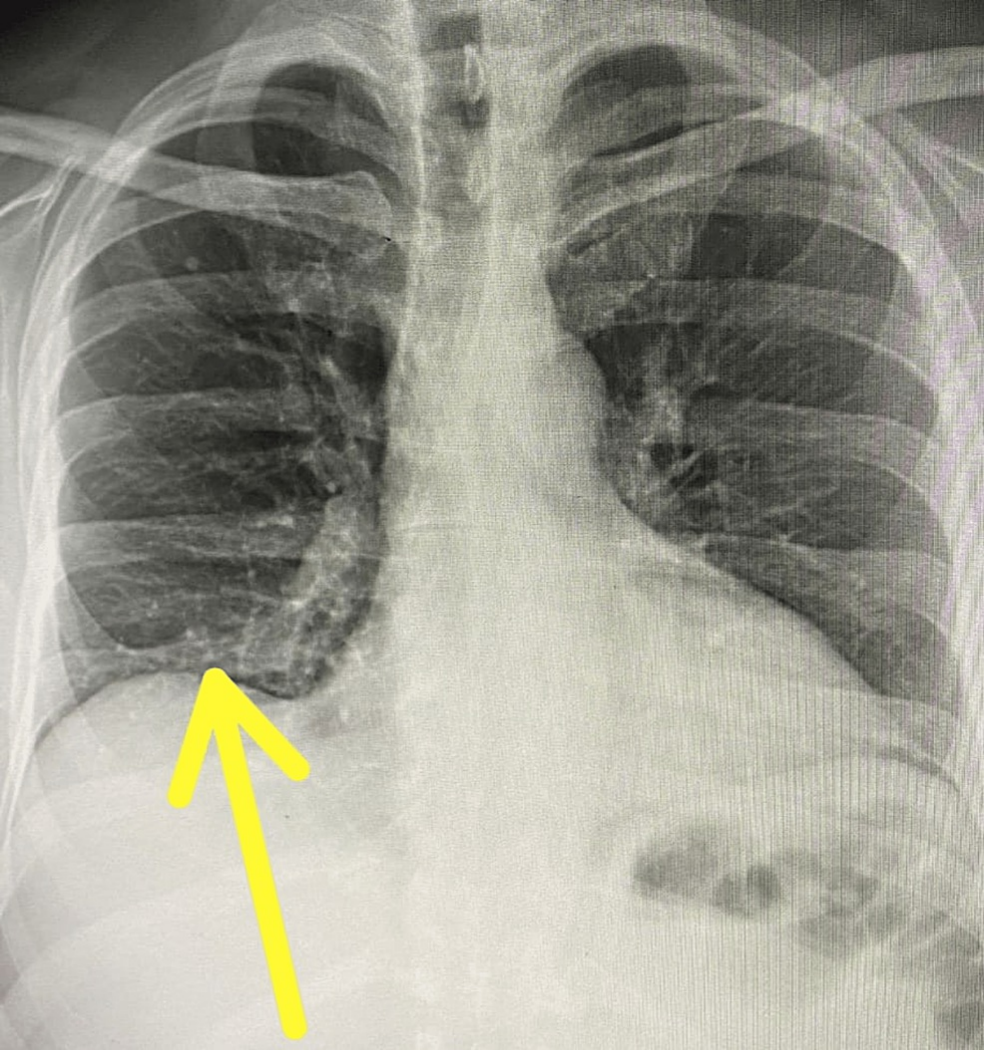
Chest X-ray image on admission The arrow shows pulmonary infiltrates.

In this presentation, the patient was doing relatively well until last fall, when he contracted COVID pneumonia with subsequent COVID-19 ICU hospitalization for two days. At that point in time, his chest X-ray showed multiple scatters of pulmonary infiltrates. He continued to have post-COVID atypical chest pain, tightness, and diaphragmatic dysfunction with severely restricted respiratory efforts (Figure 1).

This time the nasal PCR test was positive, the chest radiograph was evident, as well as the blood tests: the complete blood count, kidney function test, liver function test, and coagulation profile. However, a spirometry test was suggestive of a restrictive process (FEV1/FEV ratio > 75%) and a positive bronchodilator response (>12% increase in FEV1 or FVC and an absolute improvement of at least 200 ml). Lung volumes: moderately severe restriction (TLC 50-59%). There was a moderate decrease (40-59%) in diffusion capacity; the reduction in diffusion is proportional to reduced alveolar volume. Flow volume loops were restricted. A moderate-to-severe restriction response to the bronchodilator is noted (Table 1).

**Table 1:** Pulmonary function test spirometry readings Pre: pre-bronchodilator treatment (albuterol), Post: post-bronchodilator treatment (albuterol), pre%PRED: predicted percentage pre-treatment with a bronchodilator, Post%PRED: predicted percentage post-treatment with Bronchodilator. %PRED: predicted percentage.

Component	Ref range and units	November 24, 2020
FVC (post)	4.01–6.28 L	2.68
FEV1 (post)	3.14–4.88 L	2.28
FEV1/FVC (post)	67.83–88.20%	84.90
FEF 25–75% (post)	1.95–5.82L/s	2.97
PEF (post)	7.67–12.44 L/s	7.97
DLCO_SB	23.18–39.11 ml/(min*mmHg)	14.60
DLCO cSB	23.18–39.11 ml/(min*mmHg)	14.95
DL/VA	3.30–5.70 ml/ (min*mm Hg*L)	7.79
TLC	6.18–8.48 L	4.09
RA	1.56–2.91 L	1.83
RAW (pre)	3.06–3.06 cmH_2_O*s/L	1.64
MIP (pre)	42.75–144.47 cmH_2_O	34.77
MEP (pre)	79.43–199.16 cmH_2_O	33.24
FVC (pre)	4.01–6.28	1.49
FEV1 (pre)	3.14–4.88 L	1.12
FEV1/FVC (pre)	67.83–88.20%	75.25
FEF 25–75% (pre)	1.95–5.82 L/S	0.83
PEF (pre)	7.67–12.44 L/S	5.42
FVC (pre%PRED)	%	29
FVC (post%PRED)	%	52
FEV1 (pre%PRED)	%	28
FEV1 (post%PRED)	%	57
FEV1FVC (pre%PRED)	%	96
FEV1FVC (post%PRED)	%	108
FEF25-75 (pre%PRED)	%	23
FEF25-75 (post%PRED)	%	82
PEF (pre% PRED)	%	54
PEF (post%PRED)	%	79
TLC (%PRED)	%	56
RV (%PRED)	%	82
RAW (pre%PRED)	%	54
DLCO_SB (%pred)	%	47
DLVA (%PRED)	%	173
MIP (pre%pred)	%	37
MEP (pre%PRED)	%	24

Furthermore, electromyography was abnormal and showed a patchy chronic neurogenic process affecting the bilateral hemidiaphragm. Bilateral phrenic nerve conductions remain recordable, suggesting incomplete nerve injuries. Additionally, the thickening ratio detected by ultrasonography was in the low normal range in correlation with the patient's age.

Outcome and follow-up

Following a period of pulmonary rehabilitation, he reported persistent dyspnea. This is likely a postinfectious form of limited plexitis, which was painless because the phrenic nerve is only a motor nerve. The EMG shows that the nerve is still conducting, but at a much lower capacity than it should. Moreover, it could be recommended that he wait at least one year to see whether there is any reinnervation that might improve his lung capacity.

## Discussion

The diaphragm is the essential respiratory muscle, and its contraction is required for breathing. Any disease that interferes with diaphragmatic nerve transmission, contractile muscle function, or mechanical connection to the chest wall can cause diaphragm dysfunction. Dyspnea, exercise intolerance, sleep problems, and hypersomnia are all associated with diaphragm paralysis dysfunction, which may have an impact on survival [5]. Since the opposite diaphragm dome can compensate for this defect effectively with the aid of the external intercostal muscles, involvement of only one-half of the diaphragm does not result in significant impairment in respiratory function [1]. The most common cause of diaphragmatic paralysis is phrenic neuropathy, which can be caused by attempting to extend the neck after cardiothoracic surgery, cervical nerve root injury, neuralgic amyotrophy, or infectious agents such as herpes zoster or influenza [6].

The SARS-CoV-2 pathogen is a highly neuroinvasive infection related to neuromuscular complications such as myasthenia gravis, Guillain-Barré syndrome, and anosmia [7]. Prolonged intubation and mechanical ventilation are associated with diaphragm weakness, most likely due to critical illness, polyneuropathy, or mechanical trauma. Diaphragm failure in ventilated patients is associated with a high mortality and morbidity rate [8,9].

According to observations, body tissues infected with SARS-CoV-2 have increased angiotensin-converting enzyme 2 (ACE-2) expression; hence, the ACE-2 receptors may play an essential role in promoting viral particle invasion into neurons and other body structures, such as the diaphragm muscle, causing breathing impairment [10,11]. Notably, researchers reported that epimysial and perimysial fibrosis was more than twofold more significant in the diaphragm muscles of COVID-19-infected ICU patients compared to control ICU patients in a study on the diaphragm musculature in COVID-19-infected ICU patients [12]. Furthermore, it has been reported that lung parenchymal tissue involvement is not essential for neurogenic symptoms since one article described a COVID-19 patient who did not have lung parenchymal tissue involvement. However, later on, he developed bilateral diaphragmatic paralysis.

Additionally, because Medrinal et al. [13] documented a case report of a COVID-19 patient who acquired bilateral diaphragmatic paralysis despite having no lung parenchymal involvement, lung parenchymal involvement is not required for neurologic symptoms. In this instance, the neurologic signs of COVID-19 are most likely the result of direct viral invasion and nerve damage; nevertheless, immune-mediated events should not be ruled out [13].

A review of the published literature found that diaphragmatic impairment occurred in a COVID-19-infected patient with a past medical history of obstructive sleep apnea treated with continuous positive airway pressure (CPAP) and a mechanical ventilator. A second article by Xu et al. reveals respiratory failure caused by diaphragmatic paralysis in an obese COVID-19 patient. Shi et al. described a case report of an obese person with obstructive sleep apnea (OSA), suggesting that these features might be leading factors for developing diaphragmatic paralysis in COVID-19. A substantial burden of diaphragmatic anomalies was found in 25 COVID-19 patients treated with mechanical ventilation. Another study covers 1527 COVID-19 patients, 1.5% of whom have been diagnosed with diaphragmatic dysfunction by CT scan images. These examples differ from ours in that each subject had CPAP or ventilators, which have been identified as leading factors for developing diaphragmatic dysfunction [14,15].

In contrast, neither non-invasive nor mechanical breathing was performed on our patient. Our findings indicate that OSA and obesity may be the leading causes of diaphragmatic paralysis in COVID-19-infected patients (Table 2).

**Table 2:** The previse reported cases of the English literature of PubMed till the date The search database used was PubMed for all years up to 2022, using filters (case reports, human, and articles in English only) under the Mesh Diaphragm dysfunction and using the Diaphragm dysfunction, diaphragm paralysis, diaphragm palsy and COVID-19.

Author	Age	Sex	Diaphragm involvement	Mechanical ventilation	Diagnostic methods of the diaphragm disfunction	Other comorbidities	Treatment
Dandawate et al. [5]	56	Female	Unilateral	No	Chest fluoroscopy consistent with unilateral diaphragmatic paralysis	OSA HTN hyperlipidemia breast CA	Expectant management
Shahid et al. [6]	80	Male	Unilateral	No	NA	HTN T2DM IHD	Chest physiotherapy and incentive spirometry along with pulmonology clinic follow-up.
FitzMaurice et al. [3]	54	Male	Unilateral	Intubated and placed on lung protective airway pressure release ventilation	PFT. dynamic chest radiography	T2DM HTN OSA	Underwent surgical plication.
Boussuges et al. [7]	NA	132 patients (85 males, 47 females)	Diaphragm dysfunction of 13 patients, two of whom suffered from hemidiaphragm paralysis.	NA	Diaphragm dysfunction (DD) was detected by ultrasonography	NA	Prolonged respiratory physiotherapy led to improvement in respiratory function in most patients.
Mouir et al. [8]	58	Female	NA	NA	PFT	Obesity	NA
Law et al. [9]	NA	NA	3 months interval Chest X rays of patients post COVID-19. symptomatic patients underwent diaphragm ultrasound (n=12), pulmonary function test (n=10), muscle function test (n=6) and neurophysiology (n=5), investigating phrenic nerve function.	Of the 15 patients who have new hemidiaphragm elevation on CXR. 12 attended the clinic, 8 out of 12 patients did not receive invasive mechanical ventilation.	Ultrasound Neurophysiology	NA	Recovery from such nerve injury takes time; Summerhill et al. found return of diaphragm function could take up to 3 years, with a mean recovery time of 14.9 months. Symptomatic patients undergo surveillance for 2 years before considering onward referral to thoracic surgery for consideration of plication [11].
Lopez-Vinas et al. [10]	19	12 male 7 females	Right and left hemidiaphragm comparison between mild and sever group	NA	Sonographic and neurophysiological data	Disruption between mild and sever group in IBM, high blood pressure, diabetes mellitus, and dyslipidemia	Ventilatory rehabilitation.

Diaphragm impairment is diagnosed using static and dynamic radiological tests (especially ultrasonography), pulmonary function tests, and phrenic nerve stimulation studies. The disease's symptoms and causes will determine treatment. In asymptomatic individuals with unilateral dysfunction, diaphragm dysfunction can be treated with observation. However, in symptomatic patients with bilateral diaphragmatic paralysis, surgery (diaphragmatic plication), a diaphragmatic pacemaker, or invasive and non-invasive mechanical ventilation may be used. Non-invasive mechanical ventilation for symptomatic patients with bilateral diaphragmatic paralysis should be provided in a dedicated facility [14,16].

Weight reduction and overnight positive pressure ventilation are used to treat symptomatic individuals with unilateral diaphragmatic paralysis. Diaphragmatic fundoplication may be performed on patients who have severe or bilateral diaphragmatic paralysis [17].

## Conclusions

To conclude, COVID-19 disease cannot be considered a localized respiratory disease anymore. It is a syndromic group of multisystemic dysfunction. It could be correlated with severe non-pulmonary-related respiratory failure. However, there is a need for more literature to support the physicians as guidelines for the neurological conditions related to the COVID-19 infection. Furthermore, there needed to be more data to emphasize the prognostic factors of such a condition.
